# Dual-specificity protein phosphatase 1: A potential therapeutic target in cancer

**DOI:** 10.1016/j.isci.2025.113706

**Published:** 2025-10-04

**Authors:** Suryakant Niture, Blaine H.M. Mooers, Dee H. Wu, Matthew Hart, Jerry Jaboin, Danushka Seneviratne

**Affiliations:** 1 Department of Radiation Oncology, University of Oklahoma Health Sciences, Oklahoma City, OK 73104, USA; 2 Stephenson Cancer Center, University of Oklahoma Health Sciences, Oklahoma City, OK 73104, USA; 3 Department of Biochemistry and Physiology, University of Oklahoma Health Sciences, Oklahoma City, OK 73104, USA; 4 Laboratory of Biomolecular Structure and Function, Oklahoma University, Oklahoma City, OK 73104, USA; 5 Department of Radiological Sciences, Oklahoma University, Oklahoma City, OK 73104, USA

**Keywords:** Molecular biology, Cell biology, Radiation biology

## Abstract

Dual-specificity protein phosphatase 1 (DUSP1), also known as MAP kinase phosphatase 1 (MKP1), is a key member of the dual-specificity phosphatase family that dephosphorylates both threonine and tyrosine residues on mitogen-activated protein kinases (MAPKs). By inactivating critical MAPK signaling pathways, including extracellular signal-regulated kinase (ERK), c-Jun N-terminal kinase (JNK), and p38, DUSP1 serves as a pivotal regulator of diverse cellular processes such as proliferation, differentiation, apoptosis, autophagy, and stress responses. Emerging evidence highlights its context-dependent roles in cancer progression, where DUSP1 can function either as a tumor suppressor or promoter depending on the tumor type, stage, and tumor microenvironment (TME). Aberrant DUSP1 regulation is implicated in modulating cancer cell resistance to chemotherapy and radiotherapy, as well as facilitating immune evasion within the TME. This review provides a comprehensive overview of DUSP1, including molecular, structural, and regulatory mechanisms; its role in tumorigenesis, drug resistance, and immune modulation; and its therapeutic potential in precision oncology.

## Graphical abstract

**Figure:**
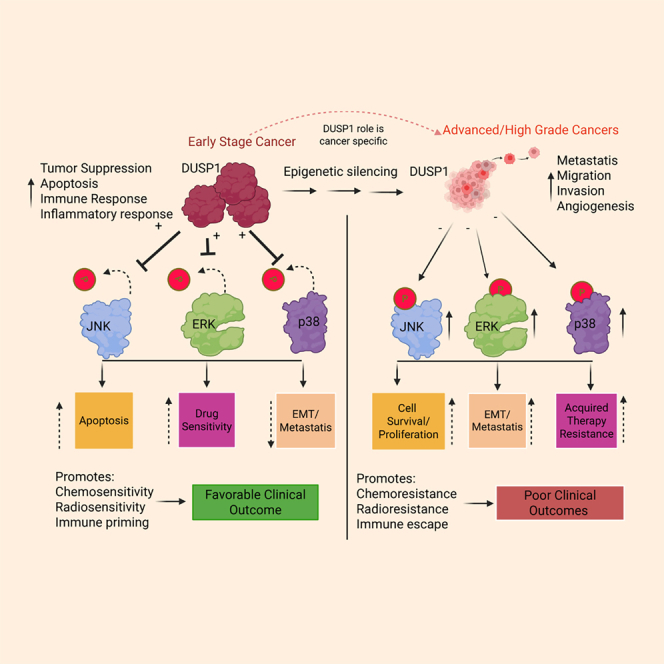

## Introduction

Despite ongoing advances in oncologic therapeutic approaches, tumor progression, tumor recurrence, and metastatic spread continue to contribute to poor patient outcomes in many malignancies. While chemotherapy remains the most commonly utilized method of cancer treatment, following prolonged use, tumors often acquire drug resistance and demonstrate limited responses.^1^ Intra-tumor heterogeneity, dysregulation of oncogenic signaling cascade/pathways, and alteration of the tumor microenvironment (TME) are the main contributors to chemoresistance and radioresistance, which create challenges to achieving therapeutic access.^2^ Several tumor-related phosphatases modulate oncogenic signaling via dephosphorylation of key factors (oncogenic and tumor suppressor proteins), contributing to tumor development, progression, or tumor growth suppression.^3^^,^^4^ Differential expression of this diverse group of tumor phosphatases modulates the TME through increasing adaptation to hypoxic conditions, reprogramming tumor metabolic pathways, and by altering stromal and immune cell interactions.^5^ Because tumor phosphatases regulate oncogenic pathways, inactivation of oncogenic phosphatases and reactivation of tumor suppressor phosphatases can modify cancer cell hallmarks such as tumor cell growth, metastasis, angiogenesis, and therapeutic sensitivity.^3^^,^^6^^,^^7^

A phosphatase subgroup known as dual-specificity phosphatases (DUSPs) belongs to the protein tyrosine phosphatase (PTP) superfamily. This phosphatase subgroup can dephosphorylate both tyrosine and threonine residues. DUSP’s family comprises MAPK phosphatases (MKPs) and atypical DUSPs, which have diverse substrates.^8^^,^^9^ In cancer, DUSPs play a key role in oncogenesis, including tumor initiation, progression, recurrence, and therapy resistance, by directly dephosphorylating MAPKs. In this review, we specifically analyze, examine, and discuss the role of the founding member of the MAP kinase phosphatase family, MKP1/DUSP1, which is responsible for inactivating the mitogen- and stress-activated MAPKs. The activation of MAPK signaling through phosphorylation is known to confer resistance to chemotherapies and targeted therapies in various cancers.^8^ Dysregulation/downregulation of DUSP1 in advanced stages of cancer disproportionately activates MAPK pathway activity, thereby impacting the response to oncologic therapies. Therefore, we need to deepen our understanding of DUSP1’s regulation, expression, modification, and role in therapy resistance. In this review, we characterize the expression pattern, structural features, and functional roles of DUSP1, while also analyzing DUSP1’s association with tumorigenesis, chemoresistance, radioresistance, and immune infiltration in the TME. We further discuss the potential for pharmacologically targeting DUSP1 in various human cancers.

## DUSP1 expression/regulation

**Figure 1:**
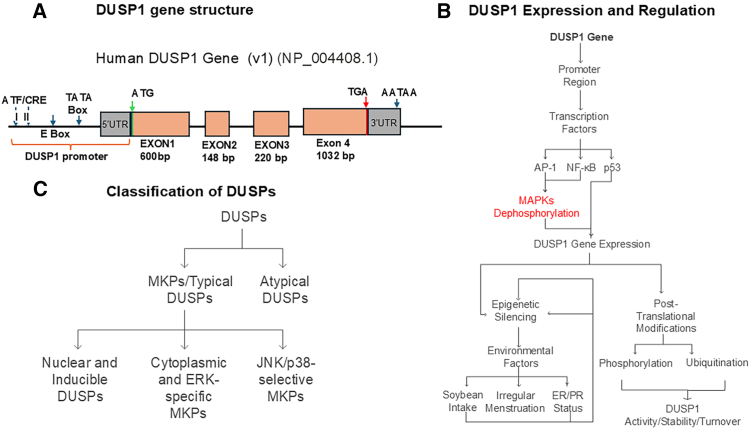
DUSP1 gene characterization and functional analysis (A) Schematic structure of the *DUSP1* gene (NP_004408.1). The *DUSP1* promoter regions binding consensus, exon, intron, and 3′UTR region are presented. (B) The transcription and post-translational regulatory factors associated with DUSP1 expression and regulation are shown. (C) Classification of typical atypical DUSPs and classification of typical DUSPs based on cellular localization and substrate specificity are presented.

The human *DUSP1* gene (ID: 1843) is also known as *CL100*, *HVH1*, *MKP1*, *MKP1*, and *PTPN10*. The gene is located on chromosome 5q35.1 between the *NEURL1B* and *ERGIC1* genes (https://www.ncbi.nlm.nih.gov/gene/1843). The *DUSP1* gene contains a full-length coding sequence of 2019 nucleotides. Gene sequence mapping reveals two conserved ATF/CRE binding sites (TGACGTCTT), an E Box (CACGTG), and a TATA box (C-ATAAAA) consensus in the promoter region. The *DUSP1* gene comprises four exons separated by three introns (400–500 bp) and a polyadenylation signal (AATAA) at 3′UTR (Figure 1A). The four exons of the *DUSP1* gene encode a 39.3 kDa (367 amino acids) DUSP1 enzyme.

The expression of DUSP1 is reported in most human tissues^9^ and is not directly regulated by a single transcription factor. Gluocorticoid receptors (GCs) at least partially control DUSP1 expression.^10^ On the other hand, *DUSP1* can be downregulated by AP1 and nuclear factor κB (NF-κB) transcription factors through the dephosphorylation of MAPKs (mitogen-activated protein kinases).^11^^,^^12^^,^^13^ Lie et al., predicted and identified several transcription factors, such as AP1, c-Jun, and ATFs, that likely bind to the upstream promoter region of *DUSP1* and regulate DUSP1 expression^11^ (Figure 1B).

Initial studies revealed that p53 binds to the third exon of the *DUSP1* gene (an atypical site of a consensus p53-binding site) and transactivates the *DUSP1* gene expression in response to different external stimuli.^14^^,^^15^ Epigenetic promoter silencing by methylation of the *DUSP1* gene affects DUSP1 protein expression. In breast cancer patients, the highest frequency of *DUSP1* promoter methylation was observed in both tumor DNA and peripheral blood leukocytes (PBLs) DNA.^16^ This trend was particularly prominent among the triple-negative breast cancer (TNBC) subtype. Soybean intake is significantly correlated with methylated *DUSP1* in ER and PR-negative patients, and irregular menstruation is correlated with methylated *DUSP1* in ER and PR-positive patients, indicating that environmental factors likely impact DUSP1 expression in cancer. Indeed, depending on the cellular context, inflammatory stimuli (tumor necrosis factor alpha [TNF-α], IL-1 cytokines), hypoxic conditions, heat shock, oxidative stress signals, and other signaling pathways can modulate transcriptional expression of *DUSP1*,^17^ while post-transcriptional modifications like phosphorylation, oxidation, and ubiquitination (Figure 1B) can also influence ultimate DUSP1protein activity/stability.^18^

## DUSPs classification

Dual-specificity phosphatases (DUSPs) are broadly categorized into 6 sub-groups: (1) mitogen-activated protein kinase phosphatases (MKPs), (2) atypical DUSPs, (3) PTEN protein phosphatases (PTENs), (4) cell division cycle 14 (CDC14) phosphatases, (5) slingshot protein phosphatases (SSHs), and (6) phosphatases of the regenerating liver (PRLs).^19^ So far, 29 mammalian DUSPs are reported and mainly categorized into two main groups (Figure 1C), (1) MKPs/typical DUSPs (11 members: DUSP1/MKP1, DUSP2, DUSP4/MKP2, DUSP5, DUSP6/MKP3, DUSP7, DUSP8, DUSP9/MKP4, DUSP10/MKP5, DUSP16/MKP7, and serine/threonine/tyrosine interacting like 1 (STYXL1) and (2) Atypical DUSPs (18 members: DUSP3, DUSP11, DUSP12, DUSP13, DUSP14, DUSP15, DUSP18, DUSP19, DUSP21, DUSP22, DUSP23, DUSP26, DUSP27, DUSP28, EPM2A glucan phosphatase (EPM2A), protein tyrosine phosphatase mitochondrial 1 (PTPMT1), RNA guanylyltransferase and 5′-phosphatase (RNGTT) and Serine/threonine/tyrosine interacting protein (STYX).^20^

The main difference between typical MKPs and atypical DUSPs is that typical MKPs consist of a CH2 domain at the N-terminal and a highly conserved DUSP catalytic domain (DUSP-CD). However, atypical DUSPs contain a highly conserved DUSP-CD domain but lack a CH2 domain at the N-terminal.^21^ In addition, typical MKPs are further classified based on their cellular localization and substrate specificity (Figure 1C), including 1) nuclear and inducible DUSPs/MKPs (DUSP1/MKP1, DUSP2, DUSP4/MKP2, DUSP5), 2) cytoplasmic and ERK-specific MKPs (DUSP6/MKP3, DUSP7/MKP-X, DUSP9/MKP4), and 3) JNK/p38-selective/specific MKPs (DUSP8, DUSP10/MKP5, DUSP16/MKP7).^22^ In this comprehensive review, we mainly focus on typical DUSP1/MKP1, a founding member of DUSPs, and characterize its potential role in cancer.

## DUSP1 molecular and functional characterization

**Figure 2:**
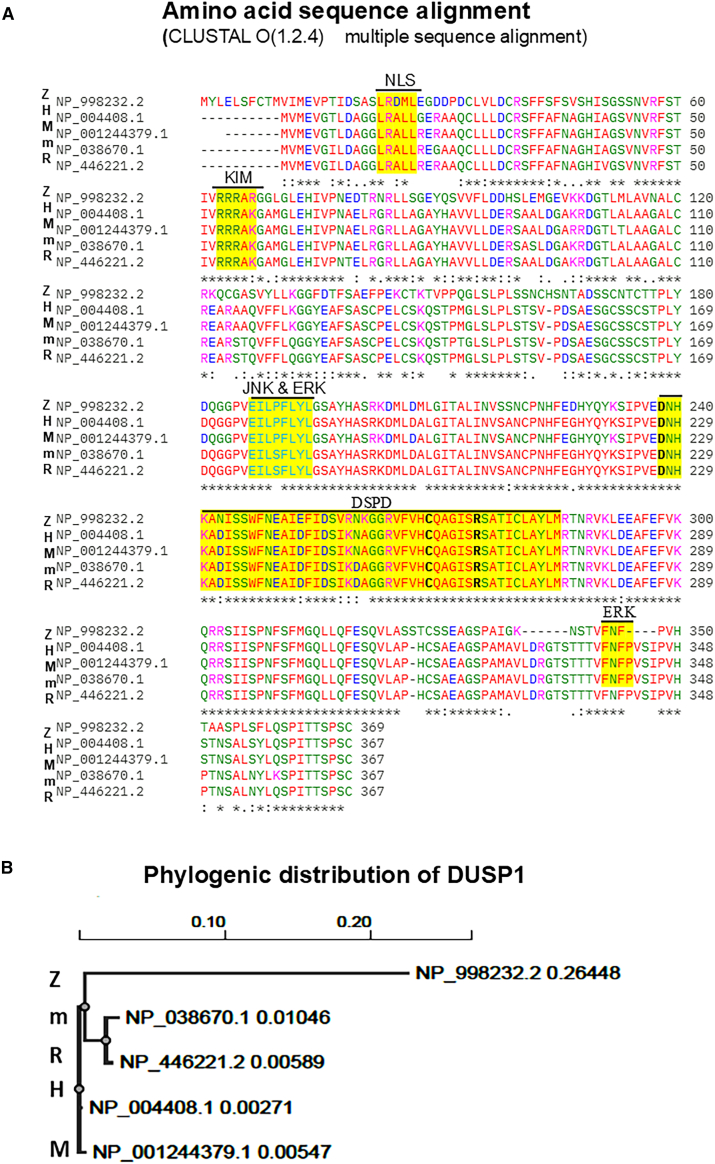
DUSP1 amino acid sequence alignment and phylogenetic analysis (A) DUSP1 amino acid sequence alignments of human (H, NP_004408.1), mouse (M, NP_001244379.1), rat (R, NP_446221.2), monkey (m, NP_038670.1), and zebrafish (Z, NP_998232.2) were performed by using EMBL-EBI Clustal Omega software tools (https://www.ebi.ac.uk/Tools/msa/clustalo/, accessed: March 2025). The catalytic domain (DSPD), substrate-binding (ERK, JNK, and KIM), and nuclear localization signal (NLS) amino acid sequences are highlighted in yellow. (B) Phylogenetic distributions of DUSP1 from human (H), mouse (M), rat (R), monkey (m), and zebrafish (Z).

By dephosphorylation of threonine and tyrosine residues of MAPKs, specifically ERK2, JNK1, and p38-alpha, DUSP1 controls MAPKs activity; however, their affinities and substrate specificities vary.^17^^,^^23^ Structurally, DUSP1 appears like a non-receptor-type protein tyrosine phosphatase and shows significant amino acid sequence similarity to a vaccinia virus Tyr/Ser-protein phosphatase encoded by gene *H1*.^24^^,^^25^ To determine the similarity of human DUSP1 with other mammalian and zebrafish encoded DUSP1, we performed amino acid sequence alignments of five species: humans, monkeys, mice, rats, and zebrafish (Figure 2A).

Between human and monkey DUSP1 protein, 99.2% identities (97.7% positives) of amino acid sequence, between mouse and rat 98.4% identities (99.5% positives), and more than 95% sequence identities/similarities were observed among these four species except zebrafish. Zebrafish DUSP1 amino acid sequence shows only ∼69% identities/similarities with the other four species. Phylogenetic analysis revealed that human and monkey DUSP1 and mouse and rat DUSP1 show the lowest phylogenetic scale compared to zebrafish, as expected (Figure 2B),^26^ suggesting that DUSP1 proteins from different mammalian species show highly conserved amino acid sequences.

**Figure 3:**
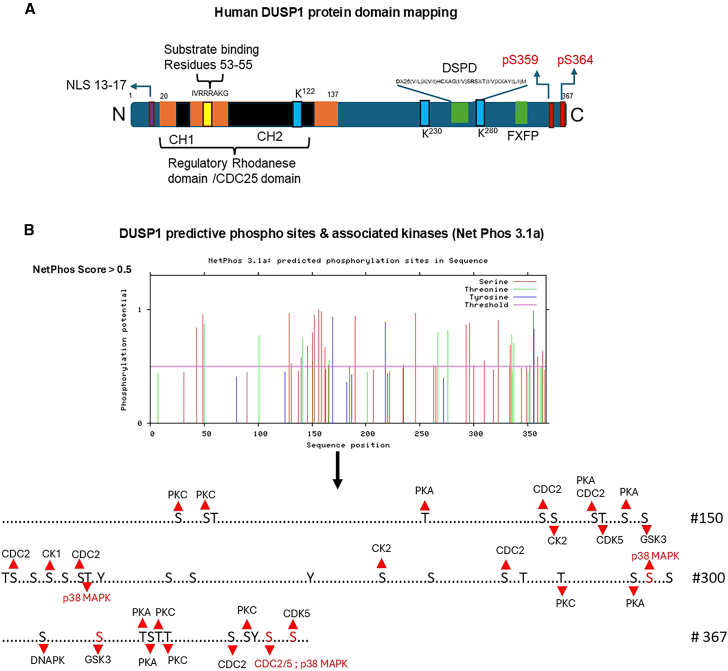
DUSP1 domains and predictive phosphorylation sites (A) DUSP1 protein key domains are represented. CH1 and CH2 CDC25 domain and substrate-binding catalytic residues of DUSP1-CD: (DSPD): **D**NHKADISSWFNEAIDFIDSIKNAGGRVFVH**C**QAGIS**R**SATICLAYLM were presented. Predicted phosphorylation, ubiquitination, SUMOylation sites, NLS, and FXFP domain in DUSP1 are shown. (B) Predicted DUSP1 phosphorylation sites from the Net Phos server 3.1a (https://services.healthtech.dtu.dk/services/NetPhos-3.1/, accessed January 2025). Locations of the predicted serine and threonine phosphorylation sites and associated kinases (with score >0.5) are presented.

We performed DUSP1 domain mapping (Figure 3A). DUSP1 consists of a CH2 domain (regulatory rhodanese domain/CDC25) near the N-terminus and a highly conserved dual-specificity phosphatase domain (DSPD) near the C-terminus (173–314 amino acids). The consensus sequence for the DSPD catalytic domain is **D**X26(V/L)X(V/I)H**C**XAG(I/V)S**R**SXT(I/V)XXAY(L/I)M (where X is any amino acid). This consensus sequence is highly conserved in human, monkey, mouse, and rat DUSPs.

In human, DUSP1, cysteine-258, arginine-264, and aspartate-227 are essential for catalysis (Figures 2A and 3A). Mechanistically, Cys258 is required for nucleophilic attack on the phosphorus of the substrate (for example, phospho-ERK2, phospho-JNK1, and phospho-p38-alpha) to form a thiol-phosphate intermediate, so Cys258 is considered the main active site residue. Arg264 binds the phosphate group of the phosphotyrosine or phospho-threonine of the substrate to promote transition-state stabilization, and aspartate-227 increases catalysis by protonating the leaving group oxygen.^27^^,^^28^ An earlier study suggests that DUSP1 lacking 53 C-terminal residues shows greater phosphatase activity, no significant change in substrate affinity or rate of catalytic reaction compared with full-length counterparts.^29^

In DUSPs, the N-terminal region is less conserved than the C-terminal region. In fact, several DUSPs lack the N-terminal CH2 domain (DUSP3, DUSP11, DUSP12, DUSP13, DUSP14, DUSP15, DUSP17, DUSP18, DUSP21, DUSP21, and others. Whereas the nuclear and inducible (DUSP1, DUSP2, DUSP4 and DUSP5), the cytoplasmic and ERK-specific DUSPs (DUSP6, DUSP7 and DUSP9), and the JNK/p38-selective/specific DUSPs (DUSP8, DUSP10 and DUSP16) consists of CDC25 homology CH2 domain.^22^

The presence of arginine-rich ^53^RRR^30^ basic kinase-interacting motifs (KIMs) at the NH2-terminal domain distinguishes the specificity of DUSPs for their MAPK targets. The KIM motif (RRRAK/R) is highly conserved in DUSP1, DUSP2, and DUSP4 and determines substrate selectivity for JNK, p38, and ERK^31^ (Figures 2A and 3A). Domain mapping studies further suggest that the C-terminal FXFP motif present in DUSP1, as well as DUSP4, DUSP6, DUSP7, and DUSP9, is involved in ERK binding,^32^^,^^33^ whereas the ^175^EILPFLYL^182^ motif found in DUSP1 mediates binding to both JNK and ERK MAP kinases.^33^ Interestingly, deletion of the LYL portion blocks the ability of the DUSP3/6 phosphatase to dephosphorylate the stress-activated protein kinase (SAPK).^34^ Indeed, these various docking sites of DUSP1 show differential affinities toward their substrates.^31^

## Post-translational and functional regulation of DUSP1

DUSP1 is an inducible nuclear phosphatase that consists of a nuclear localization signal (LRALL) at the N-terminal. This signal is also present in DUSP2, DUSP4/2, and DUSP5 (Figures 2A and 3A). DUSP1 is mainly localized in the nucleus, although other DUSPs, such as DUSP8 and DUSP16/7, possess a nuclear export signal at the C-terminus. A nuclear export signal is not present in DUSP1, so it seems impossible to shuttle DUSP1 between the nucleus and the cytoplasm.^27^ Several DUSPs, specifically JNK/p38-specific DUSP8 and DUSP16, show PEST sequences at the C-terminus involved in protein degradation and stabilization.^35^^,^^36^

DUSP1 stability is also controlled by the transient activation of ERK and activated ERK phosphorylates Ser296 and Ser323 residues of DUSP1. Ser296/323 phosphorylation triggers the interaction between DUSP1 and CUL1/SKP2/CKS1 complex (ubiquitin E3 ligase) for proteasomal degradation.^37^^,^^38^ However, ERK-mediated phosphorylation of Ser359 and Ser364 in DUSP1 near the C-terminal enhances the stability of DUSP1.^39^^,^^40^ On the other hand, the role of Ser359 and Ser364 phosphorylation in DUSP1 during ubiquitination remains unclear.^39^ Partially, krüpple-like transcription factor 5 (KLF5) regulates ERK-induced DUSP1-Ser359/364 phosphorylation that promotes breast cancer cell survival, and this phosphorylation is essential for DUSP1 protein stabilization.^41^ Phosphorylated calcium/calmodulin kinase II (CaMKII) interacts with DUSP1 and increases DUSP1 stability.^42^ In vascular smooth muscle cells, insulin stimulation enhances DUSP1 phosphorylation and stability.^43^ Conversely, when DUSP1 is destabilized or inhibited, the resulting sustained activation of JNK and p38 MAPKs leads to increased transcription of pro-inflammatory cytokines, including TNF-α and interleukin-6 (IL-6), primarily through the enhanced activation of AP1 and NF-κB transcription factors, thereby amplifying inflammatory responses and contributing to chronic inflammation-associated pathologies, including cancer progression.^44^

We recently analyzed the possible phosphorylation sites in DUSP1 proteins using the Net Phos 3.1a server (Figure 3B).^45^ There are twenty serine (that include Ser296, Ser323, Ser359, and Ser364) and seven threonine sites predicted to be phosphorylated in the DUSP1 protein, which has a significant phosphorylation score (>0.5) (Figure 3B). Many PKC, PKA, CDC2, CDK5, CK1, CK2, and p38 MAPK are presumably responsible for serine and threonine phosphorylation of DUSP1 (Figure 3B). Moreover, UniPort data search^46^ reveal three potential SUMOylation sites at Lys122, Lys230, and Lys280. Protein SUMOylation is involved in protein stabilization^47^ and this protein modification is a competitor to ubiquitination.^48^ Because DUSP1 Ser296/323 phosphorylation induces the interaction between DUSP1 and the CUL1/SKP2/CKS1 complex for ubiquitination and proteasomal degradation, it is not clear whether the same lysine residues are the targets for DUSP1 SUMOylation.^49^^,^^50^

**Figure 4:**
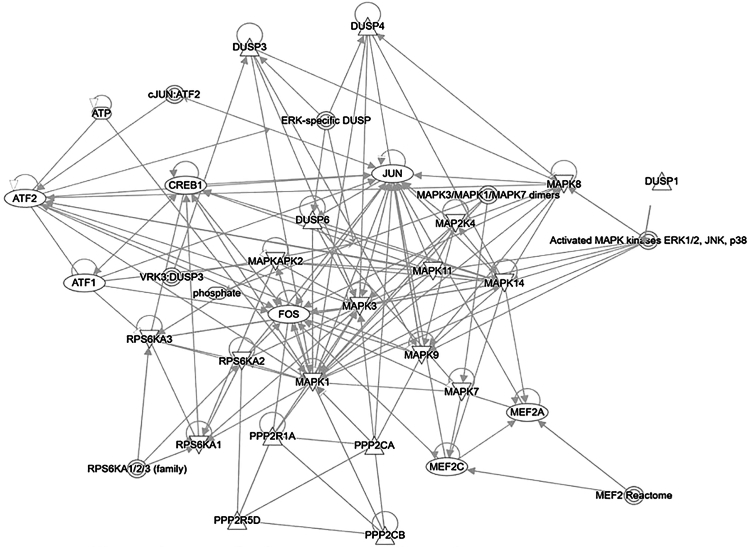
A schematic representing the interconnected functions of DUSP1 and the MAPKs DUSPs deactivate MAPKs by dephosphorylating them, which is thought to be crucial for maintaining cellular homeostasis and preventing excessive MAPK activation. DUSPs also indirectly affect CREB activation, which may regulate MAPK activity. MAPKs, such as ERK1/2, can phosphorylate CREB, leading to gene transcription. Additionally, JNK phosphorylates JUN-proto-oncogene (JUN) and FOS, which can further influence gene expression. The schematic was generated using QIAGEN’s Ingenuity Pathway Analysis (IPA, QIAGEN, Redwood City).^51^

Because DUSP1 modulates several pathways, including MAPK, ERK, EGFR, transforming growth factor (TGF)-β, and neuropathic pain-signaling, we analyzed DUSP1 interacting protein partners using QIAGEN’s Ingenuity Pathway Analysis (IPA, QIAGEN) (Figure 4). The analysis suggests that DUSP1 interacts with MAPKs, CREB1, ATF1/2, FOS, MEF2, and several other protein factors. Interactions of DUSP1 with MAPKs suggest that kinases and phosphatases regulate their activities in the loops under the influence of external stimuli. The role of DUSP1 interacting with protein partners to control DUSP1 activity, turnover, stability, and degradation in the cellular system is important from a therapeutic point of view. The detailed mechanistic role of DUSP1 interacting protein partners and their significance in cancer biology remains unknown.

## Structural aspects of DUSP1

**Figure 5:**
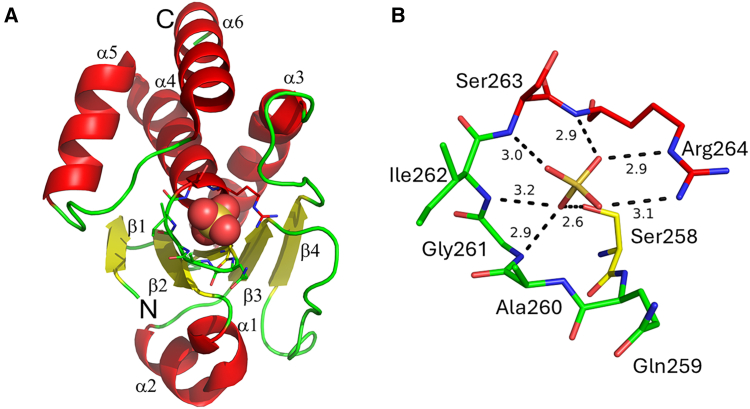
The crystal structure of the DUSP1-CD with key domains and catalytic site- PDB-ID: 6APX (A) Ribbon diagram of the DUSP1-CD. The N-terminal maltose-binding protein and the YSX1 monobody were removed from the crystal structure to improve the clarity. The secondary structure elements are colored: red for alpha helices, yellow for beta strands, and green for random coils. The active site residues, shown as sticks, interact with a sulfate, shown as spheres, that binds in a position where the phosphorus group of the phosphotyrosine on the phosphatase substrate is expected to bind. (B) The close-up view of the active site is in the same orientation as on the left and right. The distances are in angstroms and represent hydrogen bonds. The figures were made with PyMOL (The PyMOL Molecular Graphics System, version 3.1 Schrödinger, LLC).

The crystal structure of the human DUSP1 catalytic domain (DUSP1-CD) at 2.49 Å resolution was recently published.^52^ The DUSP1-CD structure is largely similar to other DUSPs’ catalytic domains, such as those of DUSP4, DUSP5, and DUSP15. Alignment of DUSP1-CD with DUSP4-CD showed the highest Z-score, and out of 143 Cα atoms of DUSP1-CD, 133Cα atoms superimposed with DUSP4-CD (which contains 144 residues), suggesting that DUSP1-CD resembles DUSP4-CD. DUSP1-CD consists of six α-helices around to the central twisted five-stranded β-sheet (Figure 5A). Two α-helices flank one side of the β-sheet, and the other four α-helices flank the other face of the β sheet.^52^ The active site DUSP1-CD consists of ^257^HCQAGISR^264^ in the phosphotyrosine binding loop, whereas Asp^227^ (Asp/D-not shown) acts as a general acid/base during catalysis (Figure 5B). In the crystal structure, a sulfate anion binds where the phosphate of the phosphotyrosine substrate is expected to bind [48]. In DUSP1-CD, an extensive hydrogen bond network exists between all four oxygen atoms of the sulfate and backbone amide nitrogen atoms of Gly^261^, Ile^262^, Ser^263^, and Arg^264^ and the side chains of Ser^258^ and Arg^264^. There are seven potential H-bonds. The geometry for potential H-bond donors from Ile^262^ and Gly^261^ are suboptimal, so they are probably partially occupied. The crystallization construct has the WT Cys^258^ mutated to Ser^258^. In this position, a Cys^258^ serves as the catalytic nucleophile in the WT enzyme.^52^^,^^53^ Designing and targeting DUSP-CD amino acid residues with inhibitors or active site-modifying reagents could be an effective strategy for controlling DUSP1 activity in the cellular context.

## Biological role of DUSP1

The expression of DUSP1 is not ubiquitous and shows widely differential tissue distributions. The DUSP1 expression was found in hematopoietic tissues, the brain, the heart, and the lungs.^54^ High DUSP1 expression is associated with multiple sclerosis, a central nervous system disease, and is related to depressive disorder.^55^^,^^56^ The expression of DUSP1 is involved in the modulation of adaptive and innate immune systems^30^^,^^57^ and obesity-related metabolic control in mice.^58^ Earlier studies suggest that disruption of the DUSP1 gene in mice does not affect the developmental phenotype, and embryonic fibroblasts and stem cells deficient in *DUSP1* indicate no change in MAP kinase regulation.^59^ Meanwhile, studies using *Drosophila* and *Arabidopsis* show that DUSP1 modulates MAP kinase activities *in vivo*. For example, in *Drosophila*, mutations in *puckered* (encoding an MKP) exacerbate developmental defects and embryonic lethality because of hyper-activation of JNK.^60^ In *Arabidopsis*, disruption of a gene that encodes an MKP and AtMKP1 become hypersensitive to genotoxic stress and the study revealed that AtMKP1 is required for maintaining the MAP kinase activity levels that contribute to genotoxic stress resistance.^61^

In human cancers, DUSP1 controls MAP kinase activity, and particularly the MAPKs ERK pathway involved in cancer development and progression.^62^ RAS and BRAF mutations, which also dysregulate MAPK signaling, leading to uncontrolled cell proliferation and drug or therapy resistance.^63^ Although DUSP1 regulated MAPKs activity in cancer, its expression varies in a variety of cancers. Specifically, low expression of DUSP1 is linked to the development and progression of these cancers, where it is associated with poorer prognosis, higher tumor burden, and reduced survival rates in patients.^64^^,^^65^ Importantly, in human cancers such as prostate, melanoma, pancreatic, glial, ovarian, testicular, salivary gland squamous cell carcinomas (SG-SCCs), and gastric cancers, DUSP1 expression is associated with cancer cell proliferation or suppression.^66^^,^^67^^,^^68^^,^^69^^,^^70^^,^^71^^,^^72^^,^^73^ The high variation in DUSP1 expression was observed in colon, prostate, liver, epithelial and advanced epithelial ovarian cancer, and bladder cancers.^74^^,^^75^^,^^76^^,^^77^ In the next section, we will review the potential regulatory role of DUSP1 in cancer and discuss the potential of DUSP1 as a pharmacologically targetable entity.

## Functional role of DUSP1 in cancer

**Figure 6:**
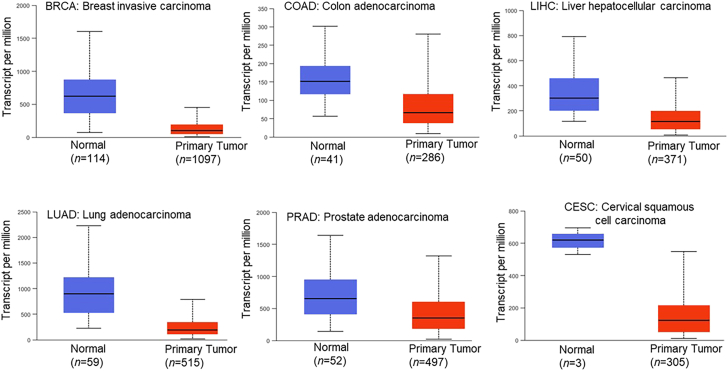
The transcriptional expression of DUSP1 in breast, colon, liver, lung, prostate, and cervical cancer was analyzed using TCGA datasets (accessed May 20, 2025) and represented (https://ualcan.path.uab.edu/index.html)

To understand the biological role of DUSP1 in tumors, we analyzed The Cancer Genome Atlas (TCGA) dataset and compared the expression of DUSP1 between normal tissues and tumors of breast, colon, liver, lung, prostate, and cervical (https://ualcan.path.uab.edu/index.html).^78^ Interestingly, TCGA data suggests that DUSP1 expression is significantly (*p* < 0.05) lower in tumors compared with adjacent normal tissues (Figure 6). However, in these patients, the overall survival was not significantly affected when DUSP1 expression was low or medium compared with high DUSP1 expression in tumors (breast cancer *p* = 0.38, colon cancer *p* = 0.13, liver cancer *p* = 0.54, lung cancer *p* = 0.59, prostate cancer *p* = 0.84, and cervical cancer *p* = 0.48). The loss or decreased expression of DUSP1 was observed in higher histological grade tumors of the prostate, pancreas, colon, ovary, bladder, gastric, and HCC,^70^^,^^74^^,^^75^^,^^77^^,^^79^^,^^80^^,^^81^ which may lead to consistent activation of MAPKs and drug resistance.^76^ In the next section, we review the functional role of DUSP1 in various cancers to understand the significance of this protein in cancer progression or suppression.

### Prostate cancer (PCa)

Prostate cancer tumors originate from the prostate gland. These tumors are generally found in older men, and DUSP1 expression is associated with PCa suppression, and an inverse correlation was reported between DUSP1 expression and activation of p38 MAPK and p65/NF-κB in human prostate cancer tissue specimens.^13^ In contrast, overexpression of DUSP1 increased DU145 PCa cell apoptosis by inhibiting TNF-α-induced p38 and JNK activation, and by inhibiting NF-κB activity through blocking p65/NF-κB nuclear translocation. Whereas specific inhibition of p38 induced a similar cell apoptosis effect compared with DUSP1 overexpression or NF-κB activity inhibition, this indicates that, rather than DUSP1, p38 regulates DUSP1-mediated cell apoptosis and NF-κB activity.^13^ The study further pointed out that high expression of DUSP1 was found in benign prostatic hyperplasia and low-grade prostatic intraepithelial neoplasia, where the levels of expression of activated p38 and p65/NF-κB are lower. On the other hand, DUSP1 levels are undetectable in high-grade prostatic neoplasia and prostatic adenocarcinoma tissues, where high expression of nuclear p65/NF-κB and activated p38 was found, suggesting that DUSP1 levels may modulate PCa development and progression differently in different stages of prostate cancer.^13^ In addition, DUSP1 knockdown increases expression of mesenchymal marker Snail and activated MAPKs, which leads to enhanced PCa cell migration and invasion, whereas DUSP1 overexpression inhibits Snail expression, cell migration, and invasion.^82^ An inverse expression correlation was observed between DUSP1 and Snail and activated MAPKs in prostate cancer tumors; high DUSP1 expression is associated with low activated JNK, ERK, and Snail levels, and the study further pointed out that the higher levels of DUSP1 expression exhibit better prognosis and extended survival in PCa patients.^82^

In small cell carcinoma of the prostate (SCCP), DUSP1 expression was lower in cancerous tissues in comparison to adjacent normal prostate tissues. DUSP1 overexpression increased cell proliferation in PC3 PCa cells by inhibition of MAPK signaling (blocking the phosphorylation of p38 MAPK), and silencing DUSP1 activated MAPK signaling and increased cell proliferation, suggesting that DUSP1 expression is associated with SCCP progression and may be a potential therapeutic target for SCCP treatment.^83^ In castration-resistant prostate cancer (CRPC), ubiquitin-specific protease 33 (USP33) was found to interact with DUSP1, reduce the DUSP1-Lys48-linked polyubiquitination, and negatively regulate JNK activation, whereas USP33 knockdown increased proteasomal degradation of DUSP1. The study further pointed out that overexpression of DUSP1 can control/reverse USP33 knockdown-induced JNK activation and cell apoptosis in docetaxel-treated prostate cancer cells, suggesting that USP33 overexpression in prostate cancer stabilized DUSP1 (by de-ubiquitination) and may contribute to docetaxel resistance.^84^

Recently, several strategies have been reported to activate or target DUSP1 in PCa cells. In PCa, several genetic and epigenetic alterations can occur that lead to disease progression. The association of PIWI-interacting RNAs (piRNAs), their genetic variant, and PCa risk targets has been studied recently.^85^ Ben et al. identified rs17201241 (a specific genetic variant) associated with increased expression of PROPER (piRNA overexpressed in prostate cancer) in PCa tumors. PROPER coupled with YTHDF2 [*N*^6^-methyladenosine (m^6^A) 5′-UTR] and YTHDF2/YBX3 (3′-UTR) promotes *DUSP1* circularization. This mRNA-looping pattern inhibits *DUSP1* translation, which promotes PCa metastasis via activation of p38. Further inhibition of PROPER expression using antagoPROPER suppresses PCa xenograft tumor growth in mice, suggesting that DUSP1 is a potential therapeutic target in PCa.^85^

A polyphenolic chemopreventive drug, resveratrol, increases the efficacy of cisplatin. Resveratrol exposure increased DUSP1 expression in androgen-independent (AR+) PCa cells, which inhibits NF-κB pathway and affects Cox-2 expression.^86^ In cooperation with cisplatin, resveratrol increases DUSP1 levels and promotes cell apoptosis, indicating that resveratrol-mediated induction of DUSP1 enhances cisplatin sensitivity of PCa cells for apoptosis.^86^

A derivative HXL131 of Matijin-Su (*N*-(*N*-benzoyl-L-phenylalanyl)-*O*-acetyl-L-phenylalanol, MTS), a phenylalanine dipeptide monomer compound isolated from Matijin (*Dichondra repens* Forst.), shows a potential inhibitory effect on cell growth, G2/M cell arrest, and migration in PC3 PCa cells.^87^ The study suggests that HXL131 exposure increased DUSP1, CYR61, TIMP1, SOD2, IL-6, SERPINE2, TNFSF9, OSMR, TNFRSF10D, and TNFRSF12A proteins that participate in PCa cell activity inhibition, whereas, the molecular docking and cellular thermal shift assay indicate that HXL131 shows strong binding affinity with DUSP1 and TNFSF9, suggesting that targeting DUSP1 by HXL131may be useful to control PCa growth and metastasis.^87^

Overall, these studies suggest that high DUSP1 expression in benign prostate tissues and at the early stages of PCa may prevent PCa incidence by controlling MAPK activity. In contrast, the loss of DUSP1 expression in high-grade PCa may exacerbate PCa angiogenesis and metastasis. Activation of DUSP1 by chemopreventive drugs at late stages of prostate cancer (PCa) will be a promising strategy for controlling PCa.

### Ovarian cancer (OC)

Several studies demonstrated that DUSP1 affects ovarian cancer (OC) progression by targeting MAPK activities, AMPK, and mTORC pathways. Higher expression of DUSP1 was observed in high-grade serous ovarian cancer (HGSOC), which severely affects progression-free survival (PFS) and overall survival (OS) in patients due to therapy resistance.^88^ Inhibition of DUSP1 by DUSPi (inhibitor) *in vitro* induced OC cell death, and the study further demonstrates that the patient-derived xenograft (PDX) HGSOC model, inhibition of DUSP1 significantly inhibited tumor progression by differential regulation of AMPK and mTORC pathways.^88^

Chondroitin sulfate N-acetylgalactosaminyltransferase 2 (CSGALNACT2) expression is downregulated in OC metastatic tissue, and stable CSGALNACT2 knockdown induced OC cell migration and invasion, whereas CSGALNACT2 overexpression reduced DUSP1 expression, which leads to increased ERK phosphorylation and subsequent suppression of OC cell migration and invasion.^89^ In addition, using an OC cohort study, the author pointed out that the CD8^+^ T cell-enriched/CSGALNACT2-high group of OC patients show worse prognosis compared with the CD8^+^ T cell-enriched/CSGALNACT2-low group. The CD4^+^ T cell-enriched/CSGALNACT2-high group was associated with a better prognosis of OC patients, indicating that CSGALNACT2 may prime different immune cell infiltration on OC tumors by modulation of DUSP1/MAPKs signaling.^89^

Similarly, Liang et al.^90^ analyzed DUSP1 expression in a clinical OC cohort; a downregulation of DUSP1 expression (mRNA and protein) was observed in OC tumors as well as the peripheral blood of OC patients. The report further suggests that the modulation of MAPK signaling and the downregulation of DUSP1 attract tumor-infiltrating T cells and cancer-associated fibroblasts (CAFs) in OC TME. High DUSP1 expression shows lower IC50 of cisplatin, paclitaxel, and gefitinib compared with low *DUSP1* expression in patients’ tumors, indicating DUSP1 not only participates in immune infiltration but also contributes to drug resistance in OC.^90^

The role of mir-141–3p/DUSP1 axis in another gynecological uterine cervical cancer (UCC) has recently been analyzed.^91^ The study demonstrated that aberrant higher expression of mir-141–3p target DUSP1 in UCC and downregulation of DUSP1 exacerbated UCC progression. Inhibition of mir-141–3p or DUSP1 overexpression suppresses the invasiveness and metastasis of UCC HeLa cells.^91^ In summary, these studies suggest that DUSP1 expression is associated with OC tumorigenesis, and by the modulation of MAPK signaling, DUSP1 may modulate immune infiltration in OC tumors. This phenomenon needs further investigation.

### Liver and gallbladder cancer

Lower DUSP1 expression was noted in hepatocellular carcinoma (HCC) tumors in comparison to normal liver tissues.^77^^,^^92^ Lower DUSP1 levels significantly correlated with high serum alpha-fetoprotein levels and were associated with increased tumor size.^77^ In HCC patients, disease-free survival rates in DUSP1-negative vs. DUSP1-positive patients are 0 and 31.0% at 5 years, respectively. After a surgical resection, the 5-year survival rate of DUSP1-negative patients is 18.2% compared with 65.5% in DUSP1-positive patients,^77^ suggesting that the expression of DUSP1 in HCC is a prominent prognostic marker for the outcome.^77^ Moreover, DUSP1 overexpression induces HCC cell apoptosis by activation of p53 and through dephosphorylation/inhibition of p38 MAPK/HSP27 signaling, indicating that reactivating DUSP1/WT-p53 could be highly beneficial in treating HCC.^92^ Upf1 (Up-frameshift 1), a nonsense-mediated mRNA decay (NMD) variant, reduces HCC tumorigenesis by increasing DUSP1 expression post-transcriptionally in the xenograft mouse model.^93^ Exposure of formosanin C (diosgenin saponin) to HCC HepG2 and Huh-7 cells suppressed cell proliferation/migration/invasion by induction of cell apoptosis, and by upregulation of DUSP1 and activation of autophagy through AMPK/ULK1/Beclin1 axis. Formosanin C suppressed HCC tumor growth in the mouse xenograft model as well.^94^ Similarly, lobetyolin (LBT) (a polyacetylene glycoside) exposure also induced HCC cell apoptosis (mitochondria-dependent) by upregulation of DUSP1. Mechanistically, DUSP1 dephosphorylates ERK1/2 and inhibits ERK1/2 activity and HCC cell growth.^95^

Like HCC, in gallbladder cancer (GBC), DUSP1 suppresses cancer cell proliferation/migration/invasion *in vitro* and inhibits GBC tumor growth and metastasis *in vivo*, when assessed in xenograft mouse models.^96^ Mechanistically, DUSP1 inhibits tumor growth by modulating pERK/MAPK-MMP2 signaling pathway and tumor metastasis by suppressing vascular endothelial growth factor (VEGF) expression, suggesting DUSP1 regulation may be an interesting therapeutic target for GBC metastasis.^96^ Collectively, these studies suggest that the lack of DUSP1 expression in advanced stages of HCC further exacerbates the disease, whereas overexpression or activation of DUSP in HCC and GBC cells suppresses cell proliferation by regulating MAPK signaling. The role of DUSP1 in non-alcoholic and alcoholic fatty liver disease and associated HCC progression is not yet clear and needs further investigation.

### Lung cancer

Gene expression profile in non-small-cell lung cancer (NSCLC) H460 cells after silencing DUSP1 revealed that the knockdown of DUSP1 modulates MAP kinase phosphatase activity, cell-cell signaling, growth factor, tyrosine kinase receptor activity, and angiogenesis.^97^ DUSP1 knockdown in H460 cells impaired Matrigel invasion *in vitro* and suppressed tumor growth and metastasis in nude mice *in vivo* due to the decrease in VEGFC production, suggesting that DUSP1 is required for angiogenesis. This correlation also exists in NSCLC patient tumors.^97^ EGFR mutation in NSCLC constitutively activates ERK1/2 activity, and both DUSP1 and DUSP4 were found to bind to ERK *in vitro* and control ERK activity. Through modulation of ERK activity, DUSP1 and DUSP4 can impact NSCLC cell survival.^98^ Moreover, the inhibition of DUSP1 decreased osimertinib resistance in NSCLC cells by enhancing cell apoptosis. Indeed, DUSP1 knockdown increased phosphorylated-JNK, ERK, and p38 MAPKs in NSCLC cells. This effect was reversed when cells were treated with the p38 MAPK inhibitor (SB203580) or when DUSP1 was overexpressed in NSCLC cells.^99^ In NSCLC, Yes-associated protein 1 (YAP1) is a main effector of the Hippo pathway that plays a significant role in osimertinib resistance. Recently, Ning et al.^100^ demonstrated that CA3 (a novel inhibitor of YAP1), in combination with Osimertinib, inhibits NSCLC cell proliferation and metastasis by induction of autophagy and cell apoptosis. YAP1, in collaboration with YY1, inhibits DUSP1 expression transcriptionally, leading to YAP1 phosphorylation and dephosphorylation of the EGFR/mitogen-activated protein kinese (MEK)/ERK pathway in osimertinib-resistant cells. YAP1 inhibition by CA3 and osimertinib exposure modulates YAP1/DUSP1/MAPKs regulatory feedback loop and cellular autophagy. The study revealed that the inhibition of YAP1 by CA3 increases DUSP1, activation of the EGFR/MAPK pathway, and induction of autophagy may enhance the efficacy of third-generation EGFR-TKI treatments for NSCLC patients.^100^

Epigenetic upregulation of DUSP1 by lncRNA CASC9 modulates gefitinib resistance in NSCLC.^101^ DUSP1 expression was controlled by lncRNACASC9 expression, which recruits histone methyltransferase EZH2 for promoter methylation and hence DUSP1 silencing, whereas ectopic overexpression of DUSP1 increased gefitinib sensitivity by inhibition of the ERK pathway.^101^

On the other hand, histone deacetylases (HDACs), an onco-protein, also control DUSP1 expression, and knockdown of HDAC induces DUSP1 expression, DISP1 upregulation inhibits EGFR signaling, and restores gefitinib sensitivity in NSCLC *in vitro* and *in vivo*.^100^ The study pointed out that increased expression of DUSP1 correlated with gefitinib-acquired resistance clinically.^102^ Moreover, a recent report suggests that DUSP1 is involved in pulmonary fibrosis by p32α inhibition and contributes to alterations in lung cellular phenotype in mouse models. Bleomycin-induced lung injury is prevented in the *DUSP1* proficient mouse model, whereas persistent lung fibrosis is observed in the *DUSP1* deficient mice.^103^

These studies suggest that DUSP1 plays a significant role in NSCLC by regulating MAPK signaling pathways, and DUSP1, in association with several other pathway factors, modulates lung cancer tumor progression, angiogenesis, invasion, metastasis, drug resistance, and apoptosis in NSCLC. DUSP1’s role in lung cancer is complex and context-dependent: Some studies suggest DUSP1 acts as a tumorigenic factor, while other studies suggest DUSP1 acts as a tumor suppressor. Further efforts are required to establish the role of DUSP1 in lung cancer.

### Colorectal cancer (CRC)

A CRC cohort study suggests that strong genetic variation in the MAPK signaling in CRC, which modulates CRC risk and patient survival after diagnosis.^104^ In colon cancer cells, ectopic inducible p53 interacts with the *DUSP1* regulatory region (a 10-bp perfect palindromic site in this), increases DUSP1 protein levels, and enhances cell death in response to nutritional stress *in vivo*.^103^ In the colorectal cancer mouse model, *DUSP1* knockout shows activation of cell adhesion, ion transport, extracellular matrix organization, response to the drug [azoxymethane/dextran sodium sulfate (AOM/DDS)], response to hypoxia, and response to toxic substances, suggesting that DUSP1 is associated with CRC development and metastasis.^105^ Moreover, dietary compounds such as butylated hydroxyanisole (BHA) and resveratrol (RSV) show colon cancer preventive activity and activation of nuclear factor-erythroid 2 related factor 2 (Nrf2).^106^ By cross-talking with NRf2, DUSP1 protects against intestinal inflammation. *DUSP1*^−/−^ and *Nrf2*^−/−^ colitis-associated tumorigenesis (CAT) mice (AOM/DDS) show similar phenotypes, i.e., significantly more tumors and higher levels of macrophage infiltration. BHA or RSV supplementation in mice suppressed CAT in wild-type mice but not in *DUSP1* ^−/−^ mice, indicating that DUSP1/Nrf2 signaling axis is required against CAT.^106^

In CRC, an aberrant expression of long noncoding RNA cyclin-dependent kinase inhibitor 2B antisense RNA 1 (CDKN2B-AS1) inhibits cell proliferation, migration, and apoptosis through the activation of MAPKs signaling. The study demonstrated that CDKN2B-AS1 binds DUSP1, the overexpression of CDKN2B-AS1 suppresses DUSP1 expression, and the overexpression of DUSP1 effectively counters the activation of MEK/ERK/p38 signaling induced by CDKN2B-AS1 overexpression.^107^ Moreover, CDKN2B-AS1 suppressed CRC tumor growth, epithelial-to-mesenchymal transition (EMT), and induced apoptosis, whereas DUSP1 promoted CRC tumor growth and EMT by suppressing cell apoptosis in the CRC mouse xenograft model. The study revealed that targeting the CDKN2B-AS1/DUSP1/MEK/ERK/p38 axis may be a promising therapeutic strategy for CRC treatment.^107^ Collectively, the role of DUSP1 in CRC is context-dependent because its interactions with tumor suppressor p53 enhance apoptosis, whereas deficiency of DUSP1 and anti-inflammatory transcription factor Nrf2 promote CAT. Activation or overexpression of DUSP1 inhibits cell proliferation, migration, and invasion in CRC cell lines. Whereas higher expression of DUSP1 downregulates cetuximab (anti-EGFR) antibody response in metastatic CRC tumors,^108^ indicating that DUSP1 expression may modulate dynamic changes in CRC-TME.

### Brain cancer

Phosphatases play an important role in solid tumors, including brain tumors, glioblastoma (GBM). As reviewed earlier, dysregulation or mutations of PTEN, PP2A, CDC25, and DUSP1 in glioblastoma modulate glioblastoma initiation, development, progression, and recurrence.^109^ The expressions of DUSP1, DUSP5, and DUSP6 were predominantly reported in pseudopalisading and perinecrotic regions of the GBM aggressive tumor, as reported in the Ivy Glioblastoma (GBM) Atlas Project (Ivy GAP) repository.^110^ Hypoxia conditions, response to dexamethasone exposure, or the chemotherapeutic agent induced DUSP1 expression in GBM cell lines and tumor-derived stem cells (TSCs).^110^ The loss of DUSP1 expression is a characteristic of TSCs and is associated with the expression of tumor stem cell markers (*ABCG2*, *NANOG*, *SOX2*, *PROM1*, and *L1CAM*) *in situ*, which indicates that DUSP1 participates in the survival of these therapeutically resistant patients associated with GBM.^110^ Moreover, chemokine CXCL12 modulates the phosphorylation of MAPKs and DUSP1 in GBM and DUSP1 decreases GBM cell death by promoting DNA repair by stabilizing the DNA repair protein RAD51 and Jun N-terminal kinase. Overexpression of DUSP1 and CXCL12 treatments increased GBM cell survival after radiation exposure.^111^

In GMB, PDGF-Rα/β, a tyrosine kinase receptor inhibitor CP-673451 induced neurite-like processes in GBM cell lines and GBM stem cells (GSCs), while reducing proliferation and invasion in 3D hyaluronic acid hydrogels. CP-673451 exposure improved the anti-tumor effects of temozolomide *in vivo* using a subcutaneous GBM xenograft mouse model. Mechanistically, CP-673451 treatment upregulates DUSP1, leading to the downregulation of p38 MAPK, which can underline the pro-differentiation effect of CP-673451 in GBM cells.^112^

Bioinformatics analysis about the gene expression of glioma-associated mesenchymal stem cells (GA-MSCs) and GBM identified 32 candidate genes primarily involved in the 1-kappa-B kinase/NF-κB and MAPK signaling pathways, both of which played critical roles in tumor survival, proliferation, and invasion. Interestingly, the study revealed that several genes, such as *FYN*, *FN1*, *G3BP1*, *FLNC*, *MYO1B*, *WLS*, and *DUSP1*, are involved in GMB progression uniquely, and targeting these genes and associated pathways could be the best approach for treating GBM tumors.^113^ Indeed, DUSP1plays a complex role in GBM, the most aggressive form of brain cancer. Depending on the context within the GBM-TME, DUSP1 can act as both a tumor suppressor and a tumor promoter.

### Breast cancer

Breast cancer (BC) remains the second leading cause of cancer death among women globally, and about one in eight women is diagnosed with BC in the United States.^114^^,^^115^ BC is a highly heterogeneous cancer and consists of several molecular subtypes, resulting in markedly varying clinical outcomes. In the United States, among the BC subtypes, 70% of cases are distributed in hormonal receptor-positive and human epidermal growth factor receptor 2-negative (HR+/HER2-), 10% are hormonal receptor-negative and HER2- (HR-/HER2-), 9% are HR+/HER2+, 4% are HR-/HER2+, and 7% are unknown cases.^114^^,^^115^ Triple-negative BC (TNBC: HR-/HER2-) is an aggressive form characterized by the absence of estrogen receptors (ERs), progesterone receptors (PRs), and HER2 expression, and shows the lowest 5-year survival rate (78%) and worse long-term prognosis compared to hormone receptor-positive (HR+) counterparts, as we reviewed recently.^116^

Notably, DUSP1 expression varies significantly across BC subtypes. In HR+/HER2-tumors, DUSP1 is often expressed at moderate levels and may function as a negative regulator of MAPK-driven proliferation. In contrast, TNBC tumors tend to exhibit dysregulated or elevated DUSP1 expressions, which have been associated with a stem-like, therapy-resistant phenotype. Recent single-cell transcriptomic analyses have revealed distinct DUSP1 expression patterns in cancer stem-like cells within TNBC tumors, highlighting its involvement in promoting survival under stress and contributing to tumor plasticity.^117^ Through a high-throughput phenotypic screening system, Shen et al. identified an organic c-glycosyl compound: aurovertin B (AVB), which shows potent anti-metastatic properties in TNBC.^118^ AVB demonstrates significant levels of metastasis suppression in MDA-MB-231, HCC1937, and 4T1 TNBC cell lines and in the orthotopic breast cancer mouse model. AVB treatments upregulate DUSP1 expression and AVB-DUSP1 interacts with activating transcription factor 3 (ATF3), which is a transcription factor for the DUSP1 transcriptional activation. Indeed, the study revealed that ATF3-DUSP1 expression and subsequent suppression of TNBC metastatic foci in response to AVB provide a promising therapeutic targeted strategy for TNBC metastasis.^118^

DUSPs such as DUSP1, DUSP4, and DUSP6 are involved in EMT and breast cancer stem cell (CSC) activity regulation. The induction of DUSP1/4/6 was observed during EMT in a PKC pathway signal-mediated EMT model.^119^ Chromatin-associated kinase PKC-θ directly regulates a subset of DUSP1/4/6 differentially with enhancer and permissive active histone post-translational modifications, suggesting that DUSPs play distinct roles in gene regulation in EMT/CSCs transitions. Knockdown of *DUSP1*, *DUSP4*, and *DUSP6* involved in the formation of CD44hi/CD24lo/EpCAM+ breast CSCs, and importantly *DUSP1* knockdown reduces CSC formation, while *DUSP4* and *DUSP6* knockdown enhance CSC formation, suggesting that DUSPs modulate EMT and CSC regulation in breast cancer differentially.^119^ In A1N4-myc human mammary epithelial cells and TNBC cells (BT-474 and MDA-MB-231), overexpression of DUSP1 decreased apoptosis and DNA fragmentation and increased drug resistance (mechlorethamine, doxorubicin, paclitaxel) due to suppression of JNK activation, whereas silencing DUSP1 enhanced drug sensitivity.^120^ These observations suggest the clinical importance of DUSP1 and the efficacy of chemotherapy. Because proteasome inhibitors are a novel class of anti-tumor compounds, and there is evidence that DUSP1 regulates sensitivity to proteasome inhibitors in breast cancer through JNK activity, targeting DUSP1 enhances the anti-tumor efficacy of proteasome inhibitors.^121^

In short, DUSP1 plays a complex role in many cancers, as stated above, in BC, higher levels of DUSP1 expression inhibit cell proliferation and promote apoptosis and act as a tumor suppressor. However, DUSP1 potentially promotes metastasis in certain contexts, and its tumorigenic role also depends on estrogen-related receptor (ERR)/PR/HER2 status of BC. Further research is needed to fully understand the complex interplay of DUSP1 with other signaling pathways and its potential as a therapeutic target in breast cancer. Some functional roles of DUSP1 in different types of cancers are summarized in Table 1.

**Table 1:** Functional and mechanistic roles of DUSP1 in human cancers

Cancer	Experimental model	DUSP1 levels	DUSP1 role	Reference
PCa	PCa cell model and human PCa tissues	downregulation	Activation p38 MAPK and p65/NF-κB in human prostate tissue specimens. Overexpression of DUSP1 impaired NF-κB activity and p38	Gil-Araujo et al.,^13^ 2014
PCa	PCa cell model and PCa clinical tissues	Knockdown	Increases expression of EMT marker Snail and activation of MAPKs that promote cell migration and invasion	Martínez-Martínez et al.,^82^ 2021
PCa	PCa cell model, male BALB/c nude mouse model, and human PCa tissues	Circularization	PROPER coupling with YTHDF2 [*N*^6^-methyladenosine (m^6^A) 5′-UTR] and YTHDF2/YBX3 (3′-UTR) inhibits *DUSP1* translation, promotes PCa metastasis, and antagoPROPER suppresses PCa xenograft tumor growth in mice	Ben et al.,^85^ 2024
PCa	PCa cell model	Activation	Resveratrol exposure increases DUSP1 expression in PCa cells, inhibits NF-κB pathway, affects Cox-2 expression, and increases the efficacy of cisplatin	Martínez-Martínez et al.,^86^ 2019
PCa	PCa cell model and Molecular docking model	Activation	Matijin-Su derivative HXL131 binds with DUSP1 and TNFSF9 to control PCa growth and metastasis	Li et al.,^87^ 2022
SCCP	SSCP cell model	Downregulation	DUSP1 silencing activates MAPK signaling and SCCP cell proliferation	Zhang et al.,^83^ 2018
HRPC	PCa cell model and human HRPC tissues	Downregulation	DUSP1 and SGK downregulation in the early stage of the HRPV tumor sample may modulate the tumorigenesis of prostate cancer	Rauhala et al.,^122^ 2005
CRPC	PCa cell model and male BALB/c nude mouse model	Upregulation	USP33 interaction stabilized DUSP1 and contributed to docetaxel resistance	Guo et al.,^84^ 2020
OC	OC cell model and PDX HGSOC model	Inactivation	DUSP1 inactivation induced OC cell death, and inhibits patient-derived xenograft HGSOC model tumor growth, by differential regulation of AMPK and mTORC pathways	Sanders et al.,^88^ 2022
OC	OC cell model and female C57BL/6 mouse model	Downregulation	Expression of CSGALNACT2 reduces DUSP1, increases ERK phosphorylation, decreases OC cell migration/invasion and modulates CD8^+^ T and CD4^+^ T immune cell infiltration in OC tumors	Ma et al.,^89^ 2024
OC	OC (OVCA) clinical tissues and molecular docking model	Downregulation	Downregulation of DUSP1 in OC tumors attracts tumor-infiltrating T cells and cancer-associated fibroblasts (CAFs) in OC TME, and high DUSP1 expression is associated with drug resistance in OC	Liang et al.,^90^ 2022
UCC	UCC tissue and cell line model	Upregulation	Inactivation of mir-141–3p upregulates DUSP1 expression and suppresses invasiveness and metastasis of UCC cells.	Liang et al.,^91^ 2024
HCC	HCC patients’ tissue model	Downregulation	Decreased levels of DUSP1 are associated with an increase in the size of HCC tumors	Tsujita et al.,^77^ 2005
HCC	HCC cell and tissue model	Upregulation	The overexpression of DUSP1 activates p53, inhibits p38 and HSP27 signaling and induces cell apoptosis	Hao et al.,^92^ 2015
HCC	HCC cell and BALB/c nude xenograft mouse model	Upregulation	Upf1-mediated increased DUSP1 expression post-transcriptionally reduced HCC tumorigenesis in the xenograft mouse model	Lee et al.,^93^ 2022
HCC	HCC cell and BALB/C nude xenograft mouse model	Upregulation	Exposure to formosanin C induced DUSP1 expression and suppressed HCC cell proliferation, migration, invasion, and tumor growth *in vivo*	Wen et al.,^94^ 2025
GBC	GBC cell, female athymic nude mouse, and human tumor model	Upregulation	DUSP1 inhibits GBC cell proliferation/migration/invasion *in vitro* and tumor metastasis by suppression of VEGF expression *in vivo*	Shen et al.,^96^ 2017
NSCLC	Lung adenocarcinoma cell model	Upregulation	The inhibition of YAP1 by CA3 increases DUSP1, activates EGFR/MAPK pathway, and induces autophagy	Ning et al.,^100^ 2023
NSCLC	NSCLC cell, nude xenograft mouse, and patient tumor sample model	Downregulation	The reduction of DUSP1 increased H460 lung cancer cell invasion *in vitro* and tumor growth in nude mice *in vivo*, and a similar correlation was observed in NSCLC patient samples	Moncho-Amor et al.,^81^ 2011
NSCLC	EGFR-mutant lung cancer cell lines model	Deregulation	EGFR mutation activates ERK1/2, and DUSP4 and DUSP1 could bind to ERK *in vitro* and control ERK activity and NSCLC cell survival	Britson et al.,^98^ 2009
NSCLC	Osimertinib-resistant NSCLC model	Downregulation	Inhibition of DUSP1 decreased osimertinib resistance in NSCLC cells by enhancing cell apoptosis	He et al.,^99^ 2025
NSCLC	NSCLC cell lines and male nude xenograft mouse model	Downregulation	Long noncoding RNACASC9 controls DUSP1 expression by promoter methylation and promotes gefitinib resistance in NSCLC	Chen et al.,^101^ 2020
NSCLC	Mouse xenograft and orthotopic lung cancer and clinical NSCLC tissue model	Upregulation	Knockdown of Histone deacetylase upregulates DUSP1, inhibits EGFR, and restores gefitinib sensitivity in NCCLC cells	Lin et al.,^102^ 2015
CRC	CRC cell model	Upregulation	Tumor suppressor p53 interacts with *DUSP1* regulatory region and induces DUSP1 expression and apoptosis in CRC cells	Liu et al.,^123^ 2008
CRC	BALB/c *DUSP1*^−/−^mouse model	Downregulation	*DUSP1* knockout in mice activates cell adhesion, ion transport, extracellular matrix organization, and CRC development and metastasis	Hammad et al.,^105^ 2021
CRC	BALB/c *Nrf2*^−/−^ and *DUSP1*^−/−^ mouse	Downregulation	DUSP1 and NRf2 deficiency modulate colitis-associated tumorigenesis in azoxymethane/dextran sulfate sodium mice model	Zheng et al.,^106^ 2019
CRC	CRC cell model and CRC xenograft mouse model	Downregulation	Long noncoding RNA cyclin-dependent kinase inhibitor 2B antisense RNA 1 (CDKN2B-AS1) binds DUSP1 and suppresses expression and CRC tumor growth, whereas overexpression of DUSP1 promotes CRC tumor growth	Pan et al.,^107^ 2021
GBM	GBM cell and GBM tumor model	Downregulation	Loss of DUSP1 is associated with GBM tumor stem cell marker expression	Mills et al.,^110^ 2017
GBM	GBM cell and GBM tissue model	Upregulation	CXCL12 activates MAPK and DUSP1 and decreases GBM cell death and DUSP1 levels, which may prevent or delay the relapses of GBM tumors during radiotherapy	Dedobbeleer et al.,^111^ 2020
GBM	GBM cell and xenograft mouse model	Upregulation	CP-673451, a PDGF-Rα/β tyrosine kinase receptor inhibitor, upregulates DUSP1, inhibits MAPKs, and induces cell differentiation in GBM cells	Lane et al.,^112^ 2022
BC	BC cell and BC tumor model	Upregulation	PKC-θ activates DUSP1/4/6 differentially and modulates EMT/CSCs transitions	Boulding et al.,.^119^2016
BC	BC cell line model	Upregulation	Overexpression of DUSP1 in TNBC reduced apoptosis and DNA fragmentation and increased drug resistance by inhibition of JNK activation	Small et al.,^120^ 2007
TNBC	BC cell and female BALB/c mice xenograft model	Upregulation	Aurovertin B exposure increases DUSP1 expression, and by interaction with ATF3, DUSP1 suppresses TNBC metastasis	Shen et al.,^118^ 2025

PCa: prostate cancer; SCCP: small cell carcinoma prostate; HRPC: hormone-refractory prostate carcinomas; CRPC: castration-resistant prostate cancer; OC: ovarian cancer; UCC: uterine cervical cancer; HCC: hepatocellular carcinoma; GBC: gallbladder cancer; NSCLC: non-small cell lung cancer; CRC: colorectal cancer; GBM: glioblastoma; BC: breast cancer; TNBC: triple-negative breast cancer.

## DUSP1 and chemoresistance

Because DUSP1 expression regulates several cellular signaling pathways in several types of cancers, the interference of DUSP1 in chemotherapy is studied in detail. High expression of DUSP1 increased chemoresistance (doxorubicin/cyclophosphamide) in breast cancer tumor cells,^124^ cisplatin resistance in osteosarcoma (OS) cells,^125^ and OC cells.^126^ Chronic adrenergic (norepinephrine) stimulation increases DUSP1 expression, which impairs paclitaxel and cisplatin chemotherapy *in vitro* and *in vivo* in ovarian cancer.^127^ Further, a correlation of DUSP1 expression and chemotherapy resistance was analyzed recently in OC.^128^ Tumor chemoresistance is frequently observed in HGSOC patients, and this second study suggests that activated (tyrosine-phosphorylated) and nuclear localized focal adhesion kinase (FAK) protects against cisplatin stress in OC cells.^129^ However, mutation of the nuclear localization sequence of FAK sensitizes OC tumors to cisplatin *in vitro* and *in vivo*. In addition, the study demonstrated that cisplatin cytotoxicity is associated with ERK-MAPK activation, and interestingly, DUSP1 inactivation (by inhibitor) enhanced both cisplatin-stimulated ERK phosphorylation and OC tumor cell death, suggesting that FAK expression and nuclear localization curtail cisplatin cytotoxicity by DUSP1-mediated noncanonical ERK/MAPK activation.^129^ Further, DUSP1 reduced chemotherapy efficacy; for example, dexamethasone (a synthetic glucocorticoid) is used to inhibit the side effects of chemotherapy in ovarian cancer patients induced DUSP1 expression and reduced chemotherapy impact.^130^

RNAi-mediated depletion of *DUSP1* increases cisplatin sensitivity in NSCLC lung cancer cells.^131^ Also, high expression of DUSP1 decreased the sensitivity of bortezomib (a proteasome inhibitor), paclitaxel (microtubule inhibitors), mechlorethamine (alkylating agents), tamoxifen, and anthracyclines like doxorubicin.^132^^,^^133^^,^^134^ In pancreatic cancer cells, high expression of DUSP1 enhances cell proliferation, migration, and invasion by upregulation of nephronectin (NPNT).^135^ Moreover, DUSP1 knockdown increases ferroptosis activator expression in pancreatic cancer cells by enhancing lipid peroxidation, and DUSP1 is involved in lipid peroxidation and ferroptotic cell death by inducing autophagy.^136^ In acute myeloid leukemia (AML), the inducible cAMP early repressor (ICER) protein, an antagonist of CREB, represses CREB-mediated gene transcription that modulates AML progression. ICER represses the expression of DUSP1 and DUSP4, which leads to activation of the p38 pathway, increased etoposide drug sensitivity and cell apoptosis.^137^ Cytarabine is used to treat high-risk myelodysplastic syndromes (MDSs), and a report suggests that the inhibition of Nrf2 by luteolin or lentiviral shRNA enhanced the efficacy of cytarabine by decreasing levels of Nrf2-targeted DUSP1 protein in myelodysplastic syndrome patients.^138^ These studies suggest that DUSP1 modulates chemotherapy resistance in several cancers and may be a potential therapeutic target for cancer treatment.

## DUSP1 and radioresistance

Radiation therapy plays a crucial role in the treatment of human cancer, and high expression of the MAPK phosphatase DUSP1 is found in many cancers, leading to radioresistance. However, the exact function of radiation in regulating DUSP1 is unclear. Conditional expression of DUSP1 in U937 (human monocyte-like histiocytic lymphoma) cells inhibit SAPK and/or p38 MAPK activity and protects cells against UV-induced apoptosis. The study suggests that DUSP1 is an upstream factor that modulates caspase activation within the apoptotic program.^139^ Further, the interplay between different MAPK pathway regulations and cell death and survival was analyzed under UV-influence in mouse embryo fibroblasts (MEFs).^140^ In response to UV radiation, activation of the p38α (but not the JNK1 or JNK2 MAPK pathways) induced DUSP1 expression, whereas MEFs deficient in *DUSP1* show activation p38α and JNK and increased sensitivity to acute UV-induced cell apoptosis, indicating that cross-talk between the p38α and JNK pathways mediated by induction of DUSP1 regulates the cellular response to UV radiation.^140^ When breast cells are exposed to radiation, DUSP1 translocates into mitochondria and prevents apoptosis by the reduction of phosphorylated active forms of JNK kinase, which can cause radioresistance in HER2-overexpressing breast cancer cells,^141^ and combined inhibition of MKP1 and HER2 enhanced cell killing in breast cancer by enhancing radiation sensitivity.^141^^,^^142^ Moreover, DUSP1 has been found to regulate DNA repair mechanisms, promoting the survival of cancer cells following radiation-induced DNA damage. Elevated DUSP1 levels after radiation exposure have been shown to enhance DNA repair by dephosphorylating H3Serin10P, which leads to epigenetic modulation and ultimately contributes to radioresistance.^76^^,^^143^ The regulation and activation of DUSP1 expression during radiation exposure/therapy and its clinical relevance remain unknown.

## DUSP1 in immune modulation

Activated MAPK kinases regulate the downstream of innate immunity and toll-like receptors (TLRs) to induce expression of the cytokines and inflammatory markers, thereby controlling MAPK activation in a timely manner that may control the immune response.^144^^,^^145^ Mitogen-activated protein kinases (MAPKs), including ERK, p38, and JNK, play pivotal roles in innate immunity by transmitting downstream signals of Toll-like receptors (TLRs). Upon activation by pathogen-associated molecular patterns, TLRs engage adaptor proteins such as MyD88 and TRIF, leading to the activation of MAPK pathways. This activation results in the transcription of pro-inflammatory cytokines and chemokines, thereby orchestrating the inflammatory response.^146^ Furthermore, the MAPK pathways are subject to tight regulation to prevent excessive inflammation. Negative feedback mechanisms, including the induction of MAPK phosphatases like DUSP1, serve to attenuate MAPK signaling, ensuring a balanced immune response.^147^

*DUSP1/2/10* deficient mice show activation of MAPK proteins, which play an essential role in local and systemic inflammation and inhibit innate and adaptive immune effector functions.^144^^,^^148^ The role of DUSP1 in the regulation of T cell activity in colorectal adenocarcinoma cells (M38) co-culture with CD8^+^ T cells was investigated. CD8^+^ T cells produced pro-inflammatory TNF-α cytokines and induced cytotoxic effects in M38 cells.^149^ DUSP1 plays a pivotal role in modulating immune responses within the tumor microenvironment, particularly influencing CD4^+^ T cells, regulatory T cells (Tregs), and natural killer (NK) cells, thereby impacting CRC progression. DUSP1 serves as a negative regulator of MAPK signaling pathways, such as p38 and JNK, which are integral to T cell activation and cytokine production. Deficiency in DUSP1 has been associated with impaired T cell responses, underscoring its significance in T cell-mediated immunity.^150^^,^^151^

Importantly, DUSP1 modulates Tumor-Associated Macrophage (TAM) activity and signaling.^44^^,^^152^ Under the influence of lipopolysaccharide (LPS), the high expression of DUSP1 dephosphorylates MAPKs such as JNK and p38, resulting in low production of cytokines TNF-α and IL-6.^44^ On the other hand, silencing of DUSP1 in HCC and oral cancer activates p38, ERK1/2, and JNK pathways that can modulate polarization of TAMs toward the M2 (anti-inflammatory) phenotype,^153^^,^^154^ and isoforms DUSP1, DUSP4, DUSP5, and DUSP6 potentially modulate TAM maturation and polarization.^152^ Although the overexpression of DUSPs in response to toll-like receptor (TLR) stimulation has been reported [119], how DUSPs regulate macrophage function in the TME is still unclear.

Many anticancer drugs regulate DUSP1 expression and immune invasion in TME; for example, sorafenib, a common drug used for HCC treatment, apart from multi-kinase inhibition, sorafenib exposure in HCC modulates anti-tumor effect via macrophages. The study suggests that microRNA-101 targets DUSP1 and downregulates DUSP1 expression, and sorafenib treatment upregulates DUSP1 expression in macrophage M2 cells, which leads to lower TGF-beta and CD206 release in HCC-TME and thus suppresses HCC progression.^155^ However, sorafenib treatment decreased miR-101 expression, increased DUSP1, and decreased TGF-β and CD206 in M2 cells, suggesting that miR-101 regulates macrophage innate immune responses by targeting DUSP1.^155^ In breast cancer, the use of Trastuzumab (a monoclonal antibody) to treat HER2-positive breast cancer decreased DUSP1 expression, activated JNK, and increased cell apoptosis. The study indicates that increased DUSP1 levels can cause anti-HER2 therapy resistance, and disruption of HER2+ DUSP1 signaling has more benefit to treat breast cancer, suggesting that DUSP1 is involved in immunotherapy resistance directly or indirectly.^141^

**Figure 7:**
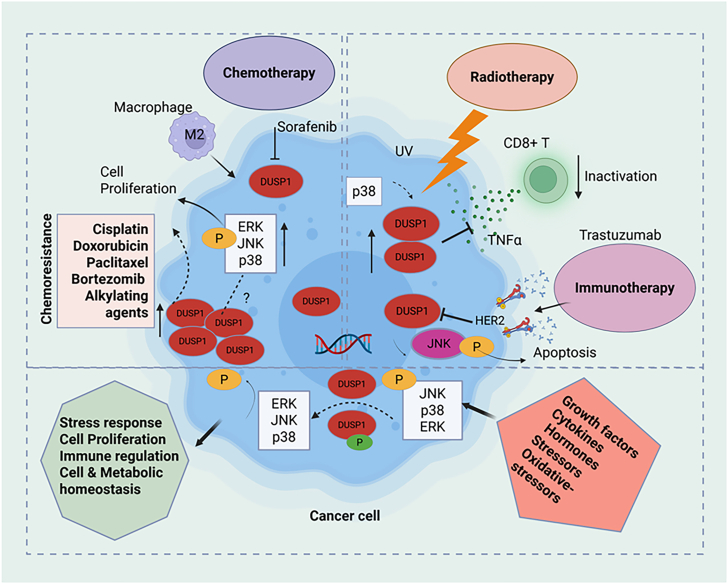
A schematic represents DUSP1’s role in chemotherapy, radiotherapy, immune modulation in TME, and drug resistance mechanisms in cancer cells By dephosphorylation of MAPKs, DUSP1 controls MAPKs’ activity and modulates cellular homeostasis. In cancer cells, targeting DUSP1 may modulate the invasion of macrophage-M2 (anti-inflammatory) in TME that could lead to chemoresistance. Radiation may regulate DUSP1 activity and inhibit CD8^+^ T cell activity. Targeting DUSP1 upstream modulators by immunotherapy, DUSP1 could induce JNK-mediated cell apoptosis. The schematic model was generated using BioRender.

Collectively, these studies suggest that the dysregulation of DUSP1 expression in different stages of cancer makes targeted therapy complicated, and DUSP1 levels modulate and affect chemotherapy, radiotherapy, and immunotherapy efficacy. DUSP1 not only induced chemotherapy resistance but also modulated radiation resistance in various cancers by targeting JNK-induced decreased apoptosis.^76^^,^^120^^,^^127^^,^^141^^,^^156^^,^^157^ Moreover, DUSP1 regulates anti-immune response by decreasing CD8^+^ T activity (by inhibiting T cell-mediated TNF-*α* production) and by activating ERK1/2, p38, and JNK pathways, which modulate maturation and polarize TAMs toward the M2 (anti-inflammatory) phenotype. Several DUSP isoforms (e.g., DUSP1, DUSP4, DUSP5, and DUSP6) potentially modulate TAM maturation and polarization from M1 to M2; thus, DUSP1 may create anti-inflammatory TME and immune escape. The functional roles of DUSP1 in the regulation of chemoresistance, radioresistance, and immune modulation are presented in Figure 7.

## DSUP1 inhibitors

Targeting DUSP1 by an allosteric inhibitor BCI ((E/Z)-2-benzylidene-3-(cyclohexylamino)-2,3-dihydro-1H-inden-1-one) modulates tumorigenesis. Several reports indicated that the inactivation of DUSP1 activity potentiates anti-tumorigenic or pro-tumorigenic impacts on tumor cell proliferation, differentiation, and survival.^158^ By targeting DUSP1/6, BCI induces apoptosis by ROS (reactive oxygen species) production and by activation of the intrinsic mitochondrial pathway in lung cancer H1299 cells.^159^ Enhance P2X7 receptor expression by downregulation of the p38 pathway in neuroblastoma cells,^160^ and exert cytotoxicity after deletion of DUSP6 and DUSP1 (CRISPR-Cas9) in neuroblastoma cells, suggesting that BCI cytotoxicity does not depend on DUSP1 or DUSP6 status in neuroblastoma cells.^161^ Moreover, targeting DUSP1/6 by knockdown or inactivation of DUSP1/6 by BCI (inhibitor) suppresses the growth of highly aggressive malignant peripheral nerve sheath tumors (MPNSTs) by induction of cell death, *in vitro* and in PDX-xenograft models.^162^ In chronic lymphocytic leukemia (CLL), the inactivation of DUSP1/6 by BCI increases hyper-activation of the MAPK signaling cascade and modulates CLL death; however, the cell death is low in malignant B cell lymphoma cells or healthy donor-derived B cells, indicating that BCI, by targeting DUSP1/6, activates an immune response in CLL,^163^ suggesting that targeting DUSP1 may be beneficial in clinical trials. Exploring the use of DUSP1 inhibitors in combination with other therapies, such as radiotherapy and immunotherapy, could potentially enhance their efficacy.

## Exploration of DUSP1 as a therapeutic target and potential side effects of DUSP1 inhibition

DUSP1 expression is dysregulated in several human cancers. The limited data available to date suggest that DUSP1 dysregulation in tumors can lead to varied outcomes, including tumorigenesis, tumor progression, chemoresistance, radioresistance, immune escape, and tumor recurrence; however, in select cases, it can also have tumor-suppressing properties. Preclinical studies indicate that therapeutically targeting DUSP1 may improve outcomes in several cancers, including pancreatic,^156^ ovarian,^88^ and melanoma.^164^ In pancreatic cancer, DUSP1 is overexpressed and promotes proliferation and tumor growth. Furthermore, DUSP1 inhibition augmented pancreatic cancer cell sensitivity to gemcitabine. Combination therapy with gemcitabine and DUSP1 increased apoptotic tumor cell death, reduced angiogenesis, and improved survival in orthotopic pancreatic mouse models.^156^ In aggressive serous ovarian cancers, DUSP1 inhibition reduced tumor cell proliferation and limited the tumor progression of patient-derived xenograft models.^88^ In primary and metastatic melanoma, DUSP1 knockdown and pharmacologic DUSP1 inhibition decreased MAPK-inhibitor resistance and sensitized melanoma cells to BRAF and MEK inhibitors.^164^ In contrast to the above noted data, in gallbladder carcinoma, increased DUSP1 expression was found to play a tumor suppressor role, inhibiting tumor cell growth, migration, and invasion in cell culture and in mouse models.^96^ Given the complexity of DUSP1 signaling as evidenced by the above studies, further investigations are required: 1) to elucidate the precise role of DUSP1 (molecular and mechanistic) in various cancer types, 2) to develop effective DUSP1 and specific inhibitors for therapeutic intervention that have minimum toxicological side effects in a preclinical setting, and 3) to assess the safety and efficacy of DUSP1 inhibitors in clinical trials.

As most cancers are treated using a combination of chemotherapy, radiation, and/or immunotherapy, when evaluating DUSP1 as a therapeutic target, it will be critical to examine the interactions between these current oncologic approaches and DUSP1 inhibition. Acquired chemotherapy resistance is an inevitable issue that eventually impacts most cancer patients. Studies show that utilizing drug combinations with non-overlapping mechanisms of action can limit the potential for drug resistance. As DUSP1 inhibition would utilize a different mechanism to kill cancer cells than traditional chemotherapies, combination therapies involving DUSP1 inhibition may reduce drug resistance and improve tumor regression.^165^ This concept is particularly strengthened by the fact that DUSP1 upregulation following traditional chemotherapies is associated with worse tumor burden and drug resistance in several malignancies and thus, DUSP1 inhibition would be expected to improve clinical outcomes. For example, during neoadjuvant chemotherapy (doxorubicin/cyclophosphamide) in breast cancer patients, increased DUSP1 levels were associated with increased residual tumor burden post-chemotherapy.^124^ Similarly, increased DUSP1 expression reduced sensitivity to cisplatin in OS and OC.^125^^,^^126^ Higher expression of DUSP1 was also associated with reduced sensitivity of many other oncologic drugs, such as bortezomib, paclitaxel, mechlorethamine, tamoxifen, and doxorubicin in breast cancer.^132^^,^^133^^,^^134^ Overexpression of DUSP1 was found to inhibit the JNK protein (which belongs to the MAPK pathway), and impede mechlorethamine, doxorubicin, paclitaxel, and doxorubicin + mechlorethamine-mediated apoptosis in breast cancer *in vivo*.^118^ In contrast, the downregulation or inactivation of DUSP1 in TNBC cells was shown to activate p38 MAPK and inhibit TNBC cell proliferation, migration, and tumor growth. DUSP1 inhibition/knockdown was found to increase cisplatin sensitivity in TNBC^166^ and NSCLC.^131^ When evaluating combination treatment approaches, assessing safety and efficacy of DUSP1 alone and in combination with standard of care oncologic approaches and coordinating the timing and delivery of DUSP1-targeted inhibitors with other cancer treatments, will be vital to achieving maximum therapeutic potential.

When considering DUSP1 as a target, one must also consider the potential side effects of DUSP1 inhibition, particularly when used in combination with chemotherapy, radiation, and immunotherapies. Assessment of acute inflammatory reaction in *DUSP1*^−/−^ mice as compared to wild-type mice demonstrated that carrageenan-induced acute paw reaction was significantly worse in mice lacking *DUSP1*.^167^ This work suggested that DUSP1 negatively regulates inflammation via inhibition of p38/MAPK signaling and inhibiting DUSP1 may promote increased acute inflammation and stress response in patients receiving anti-DUSP1 therapies. In such patients, this inflammatory effect may manifest in the form of a heightened acute immune response due to hyper-activation of immune cells such as macrophages and microglia, increased systemic cytokine production, and/or the development of autoimmune phenomena in various organ systems. Additionally, given that DUSP1 signaling protects neurons from oxidative stress, its inhibition may increase neuronal cell death and exacerbate neurodegenerative conditions such as Alzheimer’s and Parkinson’s through overactivation of the JNK/p38 pathways. In lentiviral models of Huntington disease (a devastating and incurable neurodegenerative disease), expression of DUSP1 was found to be sufficiently neuroprotective and prevented neuronal cell apoptosis.^168^ Given that DUSP1 inhibition may worsen underlying autoimmune conditions and neurological disorders, when developing clinical trials, it must be noted that cancer patients with these comorbidities may not be ideal candidates for DUSP1 targeting. The appropriate doses that will achieve cancer regression while minimizing these side effects must be assessed extensively in preclinical work and in phase I/II clinical trials. Furthermore, additional medications that may mitigate some of these undesired side effects of DUSP1 inhibition should be investigated concomitantly with DUSP1 inhibition.

Despite the promising preclinical data, there are currently no oncology-focused clinical trials that therapeutically target DUSP1. At this time, ClinicalTrials.gov only demonstrates a single study in ovarian cancer in which DUSP1 expression is being assessed. The scarcity of DUSP1-targeting clinical trials is likely due to the conflicting preclinical data about the role of DUSP1 in various malignancies and the lack of effective and therapeutically usable DUSP1 inhibitors. Therefore, it is paramount that we strengthen existing preclinical data, validate the role of DUSP1 in different malignancies, and develop safe and efficacious DUSP1 inhibitors prior to proceeding to clinical trials.

## Conclusions and future perspectives

Emerging evidence indicates that the DUSP family members, including DUSP1, are aberrantly expressed in human malignancies and may contribute to tumorigenesis and metastases. Through regulation of the MAPK (JNK, p38, and ERK1/2) signaling pathways, DUSP1 plays an important role in determining the sensitivity to a wide range of cancer therapeutic strategies. Preclinical studies indicate that DUSP1 plays a pro-oncogenic role in breast, pancreatic, ovarian, and melanoma, while serving a tumor suppressor role in gallbladder cancer. Therapeutically targeting DUSP1 has been shown to improve tumor cell death and overcome resistance to several chemotherapeutic agents.

To date, studies on DUSP1 inhibition have been confined to preclinical models, with limited clinical validation. Unfortunately, so far, no oncologic clinical trials have been carried out where DUSP1 is the sole target. A key limitation in DUSP1 targeting at this time remains selecting the appropriate cancer types that would benefit the most from DUSP1 inhibition. Despite promising preliminary data, further preclinical work must be conducted in cells and animal models to better clarify the duality of DUSP1 in various cancers and to devise strategies to mitigate the undesired side effects of DUSP1 inhibition. Additionally, while sorafenib, trastuzumab, and BCI have reduced DUSP1 activity in preclinical settings, more specific novel DUSP1 inhibitors must be developed prior to proceeding to in-human clinical trials. Clearly, characterizing the context-dependent mechanisms of DUSP1 across different malignancies and developing selective and specific DUSP1 inhibitors will be the first steps in our path to pursuing DUSP1-based precision medicine in oncology.

## Acknowledgments

We are sincerely thankful for the investigator-initiated trials grant (MDSCC309) to D.S. from 10.13039/100015308Stephenson Cancer Center, Oklahoma University, United States (NIH: P30 CA225520 [PI: R. Mannel]). B.H.M.M. was supported by NIH R01 CA242845 (PIs: J. Wu, B.H.M.M., and J. Arnet) and P30 GM145423 (PI: A. West). We also acknowledge Dr. Subhajit Ghosh for editing the manuscript and suggestions.

## Author contributions

Conceptualization, S.N. and D.S.; investigation, S.N. and D.S.; writing – original draft preparation, S.N., D.S., and D.H.W.; structural analysis, B.H.M.M.; writing – review and editing, S.N., D.H.W., B.H.M.M., D.S., M.H., and J.J. All authors have read and agreed to the current version of the manuscript.

## Declaration of interests

The authors declare no competing interests.
